# The biological activity of medical ozone in the hormetic range and the role of full expertise professionals

**DOI:** 10.3389/fpubh.2022.979076

**Published:** 2022-09-16

**Authors:** Marianno Franzini, Luigi Valdenassi, Sergio Pandolfi, Umberto Tirelli, Giovanni Ricevuti, Vincenzo Simonetti, Massimiliano Berretta, Francesco Vaiano, Salvatore Chirumbolo

**Affiliations:** ^1^International Scientific Society of Oxygen-Ozone Therapy (SIOOT), University of Pavia, Pavia, Italy; ^2^Tirelli Clinical Group, Pordenone, Italy; ^3^Department of Drug Science, University of Pavia, Pavia, Italy; ^4^Department of Clinical and Experimental Medicine, University of Messina, Messina, Italy; ^5^Department of Neurosciences, Biomedicine and Movement Sciences, University of Verona, Verona, Italy

**Keywords:** ozone, SARS-CoV-2, hormesis at cell level, hormesis effect, anti-oxidant

The recent paper by Cenci et al. (1) provided us with an overview of the biological action of medical ozone (O_3_) on infectious agents, with a particular focus on its anti-viral activity. Ozone is an allotrope of oxygen, a triatomic oxygen molecule that recently showed an ability to counteract the progress of COVID-19 and the development of severe-impact SARS-CoV-2 infection on humans (2, 3). Cenci et al. reported that the way by which ozone targets the SARS-CoV-2 infection involves a hormetic mechanism.

According to Calabrese, hormesis can be considered as a biphasic dose response mechanism characterized by stimulation from a low dose and an inhibition from the highest one (4–6). Hormesis is a paradoxical chemical phenomenon where a chemical toxicant, such as a xenobiotic, or a physical insult, which dose-dependently should inhibit biological functions, at a defined range of low doses behaves in a quite opposite way (6). In this circumstance, any dose-response curve characterized by a hormetic mechanism shows a U-shaped behavior (4–6). This would mean that a noxious chemical compound may have a beneficial or therapeutic activity within a defined range of low doses.

Therefore, hormesis can be used in pharmacological science if the chemical toxicant is dosed in a defined range of concentrations. In this perspective, medical ozone, usually administered in a calibrated oxygen-ozone mixture *via* autologous blood (30–85 μg/ml, i.e., 0.014–0.040 ppm), acts in a way completely different from the widely known gaseous ozone used for sanitization of indoor environments in different dose ranges (0.3–1.2 ppm) (1). While chemical ozone used for sanitization acts as a toxicant and directly kills microbes, ozone used in medicine (medical ozone) is employed in the hormetic range and triggers a complex network of signaling pathways leading to the activation of a cellular stress response. This response involves mitochondria, i.e., the “mitochondria associated endoplasmic reticulum membranes” or MAM, and regulates the activation of the inflammasome NLRP3, inducing a pro-inflammatory signal, and the ROS-mediated signaling toward the Nrf2-Keap1-ARE system, inducing an antioxidant and pro-survival signal (1, 7).

Cenci et al. (8) report that medical ozone acts *via* a hormetic mechanism, targeting the complex cellular machinery of the oxidative stress response, and using ozone-derived organic electrophiles, such as 4-hydroxynonenale (4-HNE) or PUFA-derived mediators such as LOPs or even cholesterol-derived oxysterols (1, 9, 10).

However, in Cenci et al., the topic appears to be particularly focused on the ability of ozone to activate a pro-inflammatory status, a consideration that is somehow contradictory with the evidence reporting medical ozone as able to suppress COVID-19 inflammation (1, 11). The role of mitochondria is particularly crucial, and Cenci et al. introduced the concept of “mito-hormesis” (1). Mito-hormesis is a mechanism where mitochondrial ROS (mtROS) and mitochondrial electrophiles (mtRES) behave as TLR-signaling molecules triggering mitochondria to modulate the macrophage's innate immune function in a hormetic way (12). Interestingly, SARS-CoV-2 hijacks and impairs host mitochondrial functions, leading to COVID-19 pathogenesis (13, 14).

The activity of ozone against SARS-CoV-2 is not directly addressed, therefore, but mediated by mtROS and mtRES (i.e., 4-HNE) in a hormetic mechanism. Despite ozone being transiently produced by activated neutrophils (15), and therefore participating in the inflammatory response, medical ozone acts *via* a hormetic mechanism, using its secondary mediators (LOPs) and accounting for a rigorous protocol of therapeutic dosages (8).

Despite many decades of protocol attempts and experimental science, which might even raise some critical opinion about initial empiricism in using medical ozone, physicians have reached a sound overview of how ozone acts on cell systems, resulting effectively in numerous contexts, such as in the SARS-CoV-2 infection and post-COVID (11, 16), in the migration and proliferation of neural stem cells (17), in multiple sclerosis (18, 19), in musculoskeletal disorders (20), in chronic fatigue syndrome (21), in retina maculopathy (22), and in knee osteoarthritis (23).

Ozone has pleiotropic properties, which are not solely confined to its simplistic anti-oxidant (24, 25) or anti-inflammatory (26) capability, despite ozone's ability to also target pro-inflammatory innate immune cells (27), but to its modulatory ability to use reactive oxygen species (ROS) as signaling molecules, rather than intracellular toxicants. This is a very important point to be emphasized.

A mitochondrial perspective of COVID-19 pathogenesis (28, 29), where the whole mitochondria homeostasis, biogenesis, and turnover, and their involvement in the complex cellular machinery of aging, survival, and metabolism (30) were recently addressed, may further elucidate ozone bioactivity in the SARS-CoV-2 infection (1).

Mito-hormesis accounts for the use of ROS as signaling molecules, which can be achieved by triggering ROS only in their hormetic range of concentrations and in the complex interplay of hypoxic/normoxic stimuli (31). The initial pro-oxidant activity of LOPs induces small amounts of H_2_O_2_, which turn into pro-survival (and anti-inflammatory) mechanisms in the cellular response to stressors (32). Therefore, all the successful strategies of medical ozone account for the ability of medical ozone employers in eliciting low doses of ROS by low calibrated doses of ozone itself. Ozone is a subtle regulatory substance, obviously, if used in a standardized and rigorous medical protocol (3, 33).

As ozone dosages, administration strategies, and methods are particularly critical, physicians using ozone therapy must be highly skilled.

The very urgent need for physicians using the oxygen-ozone (O_2_-O_3_) mediated therapy, is not only represented by implementing the number and frequency of the many educational and training courses, high scholarly and expert masters, and practical guidelines but enhancing the scientific debate within the few Scientific Committees and Societies dealing with the use of ozone in medicine and therapy (21, 33), even in COVID-19 (2, 11, 34, 35) and in post-COVID or PASC (16).

In Italy, the International Scientific Society of Oxygen Ozone Therapy (SIOOT), for example, leading the research for more than 40 years, is addressing the huge concern to sensitize physicians and caregivers on the correct use of medical ozone for therapy, with the awesome endowment of highly skilled experts, a renowned international experience and the very animated scientific debate within the activity of the same SIOOT members. Table 1 shows the recently published protocol on the use of ozone in COVID-19.

**Table 1:** SIOOT protocol for the treatment of SARS-CoV-2 infection.

**covid-19 phenotype**	**Clinical features**	**Therapy protocol**	**Other**
1	Fever, with/without respiratory symptoms, chest CT negative, normal saturimetry	No hospitalization. Home therapy with NSAIDs and ASA. 2–3 sessions of O_2_-O_3_-AHT(major, O_2_-O_3_-MAHT) per week for 2–3 weeks (40–50 μg/150–200 ml ozone in 150/200 ml blood). Sano3 bag	Ozone oil Rinozone^®^ nasal spray 2/day Ambient air sanitation (using AirKing^®^)
2	Fever-positive chest CT (few GGO) and/or low O_2_ Sat%	Hospitalization admission and follow-up 3 sessions O_2_-O_3_-MAHT per week for 3 weeks (40–50 μg/200 ml ozone in 200 ml blood). Sano3 bag	Ozone oil Rinozone^®^ spray (ozonized oil) 2/3 times per day Hyper-ozonized water to drink (2 glasses/8 h) mouth and eye rinses Ambient air sanitation (using AirKing^®^)
3	Fever-positive chest CT (multiple GGO foci) and/or low O_2_ Sat%	Sub-intensive care needed O_2_ therapy (15 L/m) 4 sessions of O_2_-O_3_-MAHT per week for 3 weeks (40–50 μg/150–200 ml ozone in 150/200 ml blood). Rectal insufflation with ozone (20–30 μg/100 ml)	Ozone oil (Rinozone^®^) nasal spray 2–3/day Hyper-ozonized water to drink (2 glasses/8 h) mouth and eye rinses Ambient air sanitation (using AirKing^®^)
4	Pre-ARDS	Pre-ARDS CPAP 1st week: 1 session of O_2_-O_3_-MAHT/day for 7 days a week (40–50 μg/200 ml ozone in 200 ml blood) 2nd week: 4 sessions of O_2_-O_3_-MAHT /week (40–50 μg/200 ml ozone in 200 ml blood) 3rd week: 3 sessions of O_2_-O_3_-MAHT/week (40–50 μg/200 ml ozone in 200 ml blood). Rectal insufflation with ozone (20 μg/100 ml)	Ozone oil (Rinozone^®^) nasal spray 2–3/day Hyper-ozonized water to drink (2 glasses/8 h) mouth and eye rinses Ambient air sanitation (using AirKing^®^)
5	ARDS very low pO_2_ Sat% (up to 35–40 mmHg) Pulmonary Interstitial syndrome	CPAP attempt (in case of WET interstitial syndrome) Intubation (in case of DRY Interstitial syndrome) 1 session of O_2_-O_3_-MAHT /day for 5 days/week (40–50 μg/200 ml ozone in 200 ml blood). Rectal insufflation (20 μg/100 ml ozone) for 4 weeks	Ozone oil (Rinozone^®^) nasal spray 2–3/day Hyper-ozonized water to drink (2 glasses/8 h) mouth and eye rinses Ambient air sanitation (using AirKing^®^

ARDS, Acute Respiratory Distress Syndrome; CPAP, continuous positive airway pressure; GGO, ground glass opacity, MAHT, major autohemotherapy.

The ability to use the correct medical practice and the best sound and reliable methodology of the therapy approach with ozone needs to be updated continuously with training courses, due to the increase in complex pathologies, such as COVID-19 and post-COVID, the ongoing difficulty in addressing complex multisystemic pathologies by current medicine, and the increase of colleagues using to oxygen-ozone therapy devices and methods, sometimes without full expertise.

As ozone is a toxicant, as reported by the same recent publication from Cenci et al., its handling is particularly burdensome, if not correctly addressed (33). The potentiality of the medical ozone in treating many complex pathologies, of immune, auto-immune, osteoarticular, and neuralgic origin, finds its ability in the fine modulation of the interplay oxidative response/inflammation, held by the mitochondria in the mito-hormetic mechanism (36). This evidence obliges physicians to use oxygen-ozone with the caution recommended by the Scientific Societies built up with this purpose (34).

Finally, from a therapeutic point of view, ozone can be successfully used independently of age and sex distribution, as the hormetic principle is a foundation of the cell survival mechanism, and therefore is perfectly working in any living situation, with particular emphasis during those processes leading to inflammation. Sensitivity to ozone is not dependent on the individual BMI, sex, race, or age. The range within which ozone is used is arranged depending on the pathology or ailment to be treated by the oxygen-ozone therapy.

Ozone therapy is a great opportunity for medical science and its use is spreading widely therefore needs further scientific insights to be made.

## Author contributions

MF: supervision, validation, and paper administration. LV: supervision and validation. SP: supervision, revision in conceptualization, and validation. UT, MB, and FV: validation. GR and VS: validation and references. SC: conceptualization, writing draft, revision, supervision, and submission. All authors contributed to the article and approved the submitted version.

## Conflict of interest

The authors declare that the research was conducted in the absence of any commercial or financial relationships that could be construed as a potential conflict of interest.

## Publisher's note

All claims expressed in this article are solely those of the authors and do not necessarily represent those of their affiliated organizations, or those of the publisher, the editors and the reviewers. Any product that may be evaluated in this article, or claim that may be made by its manufacturer, is not guaranteed or endorsed by the publisher.
